# A study of malaria vector surveillance as part of the Malaria Elimination Demonstration Project in Mandla, Madhya Pradesh

**DOI:** 10.1186/s12936-020-03517-w

**Published:** 2020-12-02

**Authors:** Ashok K. Mishra, Praveen K. Bharti, Anup Vishwakarma, Sekh Nisar, Harsh Rajvanshi, Ravendra K. Sharma, Kalyan B. Saha, Man Mohan Shukla, Himanshu Jayswar, Aparup Das, Harpreet Kaur, Suman L. Wattal, Altaf A. Lal

**Affiliations:** 1 Indian Council of Medical Research-National Institute of Research in Tribal Health (ICMR-NIRTH), Jabalpur, Madhya Pradesh India; 2 Malaria Elimination Demonstration Project (MEDP), Mandla, Madhya Pradesh India; 3 Directorate of Health Services, Government of Madhya Pradesh, Bhopal, India; 4 Indian Council of Medical Research, Department of Health Research, Ministry of Health and Family Welfare, New Delhi, India; 5 National Vector Borne Disease Control Programme, Ministry of Health and Family Welfare, New Delhi, India; 6 Foundation for Disease Elimination and Control of India, Mumbai, Maharashtra India

## Abstract

**Background:**

Understanding of malaria vector density, distribution, insecticide resistance, vector incrimination, infection status, and identification of sibling species are some of the essential components of vector control measures for achieving malaria elimination goals.

**Methods:**

As part of the malaria elimination demonstration project, entomological surveillance was carried out from October 2017 to October 2019 by collecting indoor resting mosquitoes using hand catch method. Susceptibility test was done for determining the insecticide resistance status of vector mosquito *Anopheles culicifacies* using standard protocols by the World Health Organization. The cone bioassay method was used for determining the efficacy and quality of insecticide sprayed. Mosquitoes collected from different ecotypes were identified and processed for parasite identification, vector incrimination and sibling species determination.

**Results:**

The two known malaria vector species (*Anopheles culicifacies* and *Anopheles fluviatilis)* were found in the study area, which have been previously reported in this and adjoining areas of the State of Madhya Pradesh. The prevalence of *An. culicifacies* was significantly higher in all study villages with peak in July while lowest number was recorded in May. Proportion of vector density was observed to be low in foothill terrains. The other anopheline species viz*, Anopheles subpictus, Anopheles annularis, Anopheles vagus, Anopheles splendidus, Anopheles pallidus, Anopheles nigerrimus* and *Anopheles barbirostris* were also recorded in the study area, although their prevalence was significantly less compared to the *An. culicifacies.* In 2017, *An. culicifacies* was found to be resistant to dichloro-diphenyl-trichloroethane (DDT) and malathion, with possible resistance to alphacypermethrin and susceptible to deltamethrin. However, in 2019, the species was found to be resistant to alphacypermethrin, DDT, malathion, with possible resistance to deltamethrin. The bioassays revealed 82 to > 98% corrected % mortality of *An. culicifacies* on day-one post-spraying and 35 to 62% on follow-up day-30. *Anopheles culicifacies* sibling species C was most prevalent (38.5%) followed by A/D and E while B was least pre-dominant (11.9%). *Anopheles fluviatilis* sibling species T was most prevalent (74.6%) followed by U (25.4%) while species S was not recorded. One *An.culicifacies* (sibling species C) was found positive for *Plasmodium falciparum* by PCR tests in the mosquitoes sampled from the test areas.

**Conclusion:**

Based on the nine entomologic investigations conducted between 2017–2019, it was concluded that *An. culicifacies* was present throughout the year while *An. fluviatilis* had seasonal presence in the study areas. *Anopheles culicifacies* was resistant to alphacypermethrin and emerging resistance to deltamethrin was observed in this area. *Anopheles culicifacies* was confirmed as the malaria vector. This type of information on indigenous malaria vectors and insecticide resistance is important in implementation of vector control through indoor residual spraying (IRS) and use of insecticide-impregnated bed nets for achieving the malaria elimination goals.

## Background

Malaria is a global public health problem with most of the morbidity and mortality in sub-Saharan Africa. Outside the African region, India has the highest burden of disease amongst the South-East Asian (SEAR) countries. It is a parasitic infectious disease transmitted by female *Anopheles* mosquitoes. More than one billion people are at risk of malaria [1] and despite of the significant reduction in malaria cases achieved in 2018, over 228 million malaria cases and 405,000 malaria-attributable deaths occurred worldwide in 2018 [2].

The Roll Back Malaria Partnership to End Malaria (RBM) launched the Global Malaria Action Plan (GMAP) in 2008 and Action and Investment to defeat Malaria 2016–2030 (AIM) in 2015 with the goal of reducing and eliminating malaria. The goals of the World Health Organization (WHO) Global Technical Strategy for malaria 2016–2030 (GTS) are to reduce malaria and mortality rate globally by at least 90% compared with 2015 levels, and to eliminate malaria from at least 35 countries, including India.

In 2016, India’s National Vector Borne Disease Control Programme (NVBDCP) launched a national frame-work to eliminate malaria by 2030 [3]. To complement the NVBDCP efforts in malaria elimination, the Malaria Elimination Demonstration Project (MEDP) was launched in 2017 in the tribal district of Mandla. This project is being undertaken in a public–private-partnership (PPP) model between the Government of Madhya Pradesh, Foundation for Disease Elimination and Control of India (FDEC), established by Sun Pharmaceuticals Industries limited, and the Indian Council of Medical Research (ICMR) through ICMR-National Institute of Research in Tribal Health (ICMR-NIRTH). The main objective of the project is to demonstrate that malaria elimination is feasible using proven and field-tested surveillance, case management and vector control strategies.

Prior studies from the central part of India have revealed that *Anopheles culicifacies* and *Anopheles fluviatilis* are the main vector species in this area [4–12]. *Anopheles culicifacies* was found to be responsible for causing about 60 to 65% malaria cases in rural and semi urban area in India [13] and *An. fluviatilis* was also incriminated as an efficient vector [10–12]. *Anopheles culicifacies* is the complex of five sibling species i.e., A, B, C, D and E [14, 15] with biological variations in all sibling species. These species differ in their role in malaria transmission [6, 16] and insecticide resistance [6, 17–21].

Government of India (GOI) introduced Indoor Residual Spraying and insecticide impregnated mosquito nets as the main intervention strategy for malaria vector control. Subsequently, it was observed that *An. culicifacies* have developed resistance to Dichloro-diphenyl-trichloroethane (DDT) [22, 23], deltamethrin [24], Benzene Hexachloride (BHC) [25] and even to pyrethroids [26]. This entomologic surveillance study was conducted as part of the Malaria Elimination Demonstration Project (MEDP) project, with a goal to identify the vector species and to ascertain relative abundance, insecticide efficacy, role in malaria transmission and their behaviour.

## Methods

**Fig. 1:**
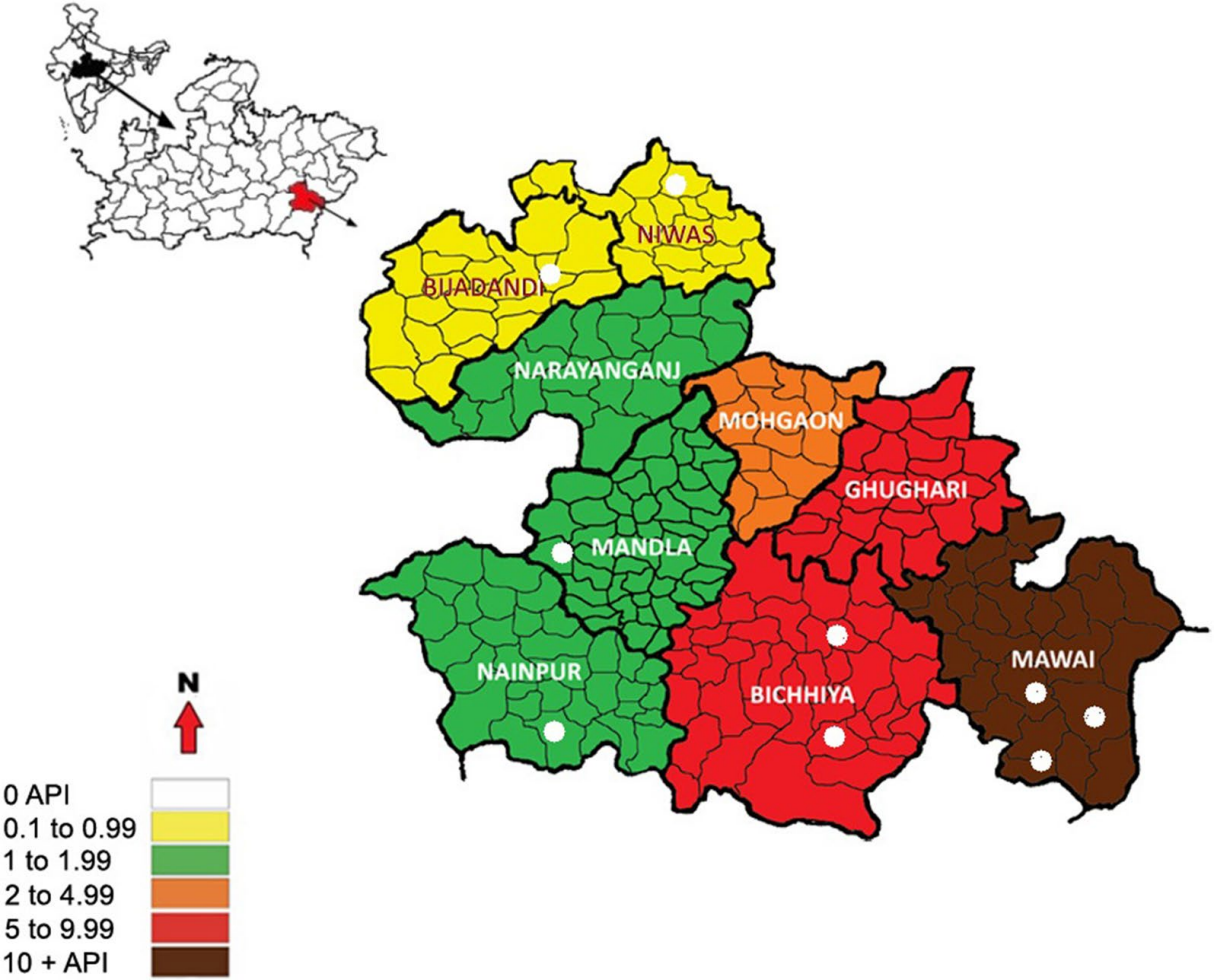
Map of Mandla district showing (highlighted in white colour) the location of mosquito collection sites

Study area description: Mandla, a tribal district is in the centre of Madhya Pradesh marked with valleys, hills and thick dense forest at altitude of 450–950 M, (23° N latitude, 80° 10′ E longitude) is the site of the project. Narmada River flows through the district which provides the breeding sites for anophelines. The area of the district is about 8771 km^2^, consisting 9 blocks (Fig. 1) with total 1.168 million population (projection for 2016 based on 2011 census data). The weather is categorized as monsoon (June–August), post monsoon (September–November), winter (December–February) and summer (March–May).

As per NVBDCP data, the malaria burden in 2015 was high (API: 3.52), while a significant reduction was observed in 2019 (API: 0.17), (Table 1). Since 2017, the district’s malaria programme used alphacypermethrin 5% in IRS twice a year in areas with Annual Parasite Incidence (API) of 1 to 4.99. The LLINs were distributed in areas with API of 5 and above in 2017, and subsequently in areas with API of more than 2 in 2019. Neither IRS nor LLINs were provided in areas which had less than 1 API. In these areas, community was informed to use the personal protection measures, such as mosquito coils, anti-mosquito ointments. IRS and LLIN distributions were done as part of routine government vector control activity through the District Malaria Office with supportive supervision provided by the MEDP staff.

**Table 1:** Malaria Burden (2015–2019) in Mandla district, Madhya Pradesh

Year	Population	BSE	Total positive	Pv	Pf	SPR	API	ABER	Death
2015	1,140,367	265,726	4018	972	3046	1.51	3.52	23.30	0
2016	1,163,173	225,001	1431	425	1006	0.63	1.23	19.34	0
2017	1,186,436	180,786	435	168	267	0.24	0.36	15.24	0
2018	1,204,233	287,461	330	101	229	0.11	0.27	23.87	0
2019	1,181,493	295,190	196	75	121	0.06	0.16	24.98	0

*BSE *Blood Slide Examined, *PV Plasmodium vivax*, *PFPlasmodium falciparum*, *SPR *Slide positive Rate, *API *Annual Parasite Incidence, *ABER *Annual Blood Slide Examination Rate

In this study, villages for entomological surveillance were selected in three category areas on the basis of API (2015) i.e. < 1(category A), 1.0 to 4.99 (category B), > 5 (category C). Three villages in each category encompassing different terrains viz., plain, forest and foothill were selected for mosquito sampling. The study was carried out between October 2017 to October 2019 by visiting study area once in every 3 months to cover all the seasons.

### Mosquito sampling

Anopheline mosquitoes resting indoors and outdoors were collected during every field visit from all nine study villages (3 villages in each API category). The mosquito specimens were collected by a team of four insect collectors with a flashlight and mouth aspirators during early morning (0600–0800 h) from 4 human dwellings and 4 cattle sheds located in different parts of the villages [27]. The same team was deployed to catch mosquitoes in each study village. Mosquitoes collected from different localities were kept in separate test tubes and labeled with location, village name, date and time of collection and brought to the field laboratory for identification and further processing for vector incrimination and sibling species determination.

### Insecticide resistance status

Insecticide resistance status of *An. culicifacies* (major malaria vector) was ascertained during the study period. Susceptibility tests on adult *An. culicifacies* were conducted following standard WHO procedures [28]. Wild-caught mosquitoes, preferably blood-fed female mosquitoes, were collected from different resting sites (indoors-human dwellings/cattle sheds) and [27] and identified based on morphological characters [29]. The collected mosquitoes were brought by temperature controlled vehicle to the laboratory (about 10–20 km away from field) for testing in cloth cages wrapped with wet towel to maintain humidity. Female mosquitoes were exposed in replicates to the WHO impregnated papers with specified discriminating dosages of the insecticides (DDT: 4%, malathion: 5%, deltamethrin: 0.05% and alphacypermethrin 0.05%), respective insecticide controls for comparison (two replicates) for one hour and mortality was recorded after 24-h holding. The tests were repeated within 2 or 3 days in different villages and different terrains. Cartons with wet towels at the bottom were used to conduct the tests to maintain the ambient temperature of 25 ± 2 °C and RH of 80 ± 10% in the field laboratory [30]. Mortality after 24 h of holding period was recorded [31].

Percent mortality was calculated separately for the test and control replicates using the formula-

$$\% {\text{ Observed mortality}}\, = \,{\text{Number of dead mosquitoes}}\; \times \;{1}00/{\text{Number of mosquitoes tested}}$$

If the mortality in control replicates is between 5 and 20%, the test mortality was corrected with the control mortality using Abbott's formula [32]. In case, the mortality in the controls exceeds 20%, the test was discarded.

$$\% {\text{ Corrected mortality}}\, = \,\left( {\% {\text{ Test mortality}}\;{-}\;\% {\text{ Control mortality}}} \right)\; \times \;{1}00/({1}00\;{-}\;\% {\text{ Control mortality}})$$

According to the WHO criteria [31], if the mortality of mosquito species on exposure to the diagnostic dosage of a given insecticide is 98 to 100%, it is designated as ‘susceptible’, if mortality is < 90%, it is designated as ‘confirmed resistance’, and possible resistance if the mortality is between 90 and 98%.

Cone bioassays were carried out during IRS months (October 2017, July, October 2018 and July and October in 2019) to assess the efficacy of insecticide used in IRS programme and also to assess the quality of IRS on different sprayed surfaces in the villages. The tests were done in 3 villages of 3 CHCs each on the basis of the availability of houses sprayed on day-one and on or after day-30 of the spray. The houses having different sprayed surfaces were selected for cone bioassays. Two unsprayed houses were selected for control. The bioassays were done on day-one and day-30 post-spraying with WHO cones [27, 33] using field collected *An. culicifacies* from unsprayed area due to their availability in sufficient numbers. A total of 10 fed-female mosquitoes were exposed to sprayed surfaces for 30 min, and kept in paper cups covered with net. These mosquitoes were given 10% glucose solution soaked in cotton wool for the maintenance of 25 ± 2 °C temperature and 80 ± 10% moisture. Percent mortalities were calculated from the total number of live and dead mosquitoes by the Abbott’s formula [32].

### Processing for vector incrimination

Mosquito species of *An. culicifacies* and *An. fluviatilis* collected during the study period were separated into head thorax and abdomen parts and dried. These mosquitoes were kept in 1.5 ml micro tubes and sent to laboratory for molecular analysis of parasite using Polymerase Chain Reaction (PCR) method. They were categorized viz., species and habitats with respect to study villages. Genomic DNA was extracted from mosquito heads and thoraces by the method described by Coen et al. [34]. Pools of genomic DNA from 10 samples were prepared and Nested PCR was performed for detection of malaria parasites from the extracted DNA with the primers described by Snounou et al*.* [35].

Sibling species identification of *An. culicifacies* and *An. fluviatilis* mosquitoes was analysed using allele-specific PCR. The identification of *An. culicifacies* was done as reported by Singh et al. [36] and Goswami et al*.* [37]*.* For *An. fluviatilis,* it was done as reported by Singh et al*.* [38]. PCR reaction mixture was prepared using 200 μM of each dNTP, 1.5 mM MgCl2, 1 × PCR buffer and 1 unit of taq DNA polymerase. The cycling conditions included initial denaturation at 95 °C for 5 min, followed by 35 cycles each of denaturation for 30 s at 95 °C, annealing for 30 s at 50 °C and extension for 1 min at 72 °C, and then final extension at 72 °C for 7 min.

### Ethical clearance

Ethical clearance was obtained from the Institutional Ethics committee (IEC) of ICMR–National Institute of Research in Tribal Health (ICMR-NIRTH) on 16th March 2017 bearing reference no. 201701/10. A verbal informed consent for mosquito collection was taken from the residents of the households.

### Data management and analysis

The data was entered in data entry software designed on CS-Pro 7.0 platform and data analysis were done with Statistical Package for Social Sciences (SPSS) v20.0 by IBM.

## Results

Mosquito resting collections: During indoor resting collections, 9 anopheline species were collected of which *An. culicifacies* (56.0%), *Anopheles subpictus* (18.0%), and *Anopheles annularis* (15.8%) were found to be the most abundant species (Table 2). The other anopheline species viz., *An. fluviatilis*, *Anopheles vagus*, *Anopheles pallidus*, *Anopheles barbirostris*, *Anopheles nigerrimus* and *Anopheles splendidus*, were found in small numbers.

**Table 2:** Relative abundance of indoor resting anophelines (per man hour) in different areas of Mandla District, Madhya Pradesh

Anopheline species	Village of category A (< 1 API)			Village of category B (1–5 API)			Village of category C (> 5 API)			Total in District		
	Nos.	%	MHD	Nos.	%	MHD	Nos.	%	MHD	Nos.	%	MHD
*An. culicifacies*	743	63.45	14.29	590	53.35	11.35	473	49.74	9.10	1806	55.95	11.58
*An. fluviatilis*	22	1.88	0.42	10	0.90	0.19	39	4.10	0.75	71	2.20	0.46
*An. subpictus*	181	15.46	3.48	200	18.08	3.85	199	20.93	3.83	580	17.97	3.72
*An. annularis*	158	13.49	3.04	194	17.54	3.73	159	16.72	3.06	511	15.83	3.28
*An. vagus*	15	1.28	0.29	7	0.63	0.13	19	2.00	0.37	41	1.27	0.26
*An. splendidus*	14	1.20	0.27	11	0.99	0.21	15	1.58	0.29	40	1.24	0.26
*An. palidus*	24	2.05	0.46	75	6.78	1.44	40	4.21	0.77	139	4.31	0.89
*An. nigerrimus*	4	0.34	0.08	2	0.18	0.04	4	0.42	0.08	10	0.31	0.06
*An. barbirostris*	10	0.85	0.19	17	1.54	0.33	3	0.32	0.06	30	0.93	0.19
*Total Anopheles*	1171		22.52	1106		21.27	951		18.29	3228		20.69

*Nos* Number, *MHD* Per Man Hour Density, *API* Annual Parasite Incidence

No consistent pattern was seen in the number of anopheline mosquitoes collected throughout the year and month-to-month variation in the number of mosquitoes was common (Table 3). *Anopheles culicifacies* was found to be the predominant species followed by *An. annularis* and *An. subpictus* during (Table 2) during 156-man hours efforts. Outdoor resting mosquito collection were also undertaken, but we were unable to collect any mosquito and therefore, after one year of the study, the outdoor collection was discontinued.

**Table 3:** Month wise and category wise malaria vector composition and man hour density in Mandla district, Madhya Pradesh

Month	Category of villages	Hrs Spent	*An. culicifacies*			*An. fluviatilis*			Total *Anopheles*	
			Nos	%	MHD	Nos	%	MHD	Nos	MHD
October-17	A (< 1 API)	4	50	39.06	12.50	0	0.00	0.00	128	32.00
	B (1–5 API)	4	37	22.02	9.25	0	0.00	0.00	168	42.00
	C (> 5 API)	4	42	28.77	10.50	2	1.37	0.50	146	36.50
	Total	12	129	29.19	10.75	2	0.45	0.17	442	36.83
February-18	A (< 1 API)	6	58	60.42	9.67	1	1.04	0.17	96	16.00
	B (1–5 API)	6	86	78.90	14.33	5	4.59	0.83	109	18.17
	C (> 5 API)	6	44	47.31	7.33	21	22.58	3.50	93	15.50
	Total	18	188	63.09	10.44	27	9.06	1.50	298	16.56
May-18	A (< 1 API)	6	21	87.50	3.50	0	0.00	0.00	24	4.00
	B (1–5 API)	6	39	86.67	6.50	0	0.00	0.00	45	7.50
	C (> 5 API)	6	45	90.00	7.50	0	0.00	0.00	50	8.33
	Total	18	105	88.24	5.83	0	0.00	0.00	119	6.61
July-18	A (< 1 API)	6	141	62.67	23.50	0	0.00	0.00	225	37.50
	B (1–5 API)	6	139	53.88	23.17	0	0.00	0.00	258	43.00
	C (> 5 API)	6	120	57.69	20.00	0	0.00	0.00	208	34.67
	Total	18	400	57.89	22.22	0	0.00	0.00	691	38.39
October-18	A (< 1 API)	6	106	73.61	17.67	3	2.08	0.50	144	24.00
	B (1–5 API)	6	109	68.99	18.17	1	0.63	0.17	158	26.33
	C (> 5 API)	6	58	45.31	9.67	6	4.69	1.00	128	21.33
	Total	18	273	63.49	15.17	10	2.33	0.56	430	23.89
January-19	A (< 1 API)	6	98	78.40	16.33	2	1.60	0.33	125	20.83
	B (1–5 API)	6	29	53.70	4.83	3	5.56	0.50	54	9.00
	C (> 5 API)	6	5	15.63	0.83	4	12.50	0.67	32	5.33
	Total	18	132	62.56	7.33	9	4.27	0.50	211	11.72
May-19	A (< 1 API)	6	26	44.07	4.33	0	0.00	0.00	59	9.83
	B (1–5 API)	6	25	51.02	4.17	0	0.00	0.00	49	8.17
	C (> 5 API)	6	18	38.30	3.00	0	0.00	0.00	47	7.83
	Total	18	69	44.52	3.83	0	0.00	0.00	155	8.61
July-19	A (< 1 API)	6	199	75.95	33.17	0	0.00	0.00	262	43.67
	B (1–5 API)	6	90	60.40	15.00	0	0.00	0.00	149	24.83
	C (> 5 API)	6	100	58.14	16.67	0	0.00	0.00	172	28.67
	Total	18	389	66.72	21.61	0	0.00	0.00	583	32.39
October-19	A (< 1 API)	6	44	40.74	7.33	16	14.81	2.67	108	18.00
	B (1–5 API)	6	36	31.03	6.00	1	0.86	0.17	116	19.33
	C (> 5 API)	6	41	54.67	6.83	6	8.00	1.00	75	12.50
	Total	18	121	40.47	6.72	23	7.69	1.28	299	16.61
Total	A (< 1 API)	52	743	63.45	14.29	22	1.88	0.42	1171	22.52
	B (1–5 API)	52	590	53.35	11.35	10	0.90	0.19	1106	21.27
	C (> 5 API)	52	473	49.74	9.10	39	4.10	0.75	951	18.29
G Total		156	1806	55.95	11.58	71	2.20	0.46	3228	20.69

*Hrs* Hours, *Nos* Number, *MHD* Per-Man Hour Density, *API *Annual Parasite Incidence

The per man per hour density of *An. culicifacies* was lowest (3.8 ± 6.7) in May 2019 and highest (22.2 ± 1.93) in July 2018 which is statistically significant (p < 0.001). The density of *An. fluviatilis,* the other known vector of malaria, was highest in February 2018 (1.5), though small numbers were caught in every visit except in July 2018, May 2019 and July 2019. The overall *An. culicifacies* density in 2017 was 10.75 ± 1.6, which slightly increased in 2018 (13.4 ± 7.0) and declined significantly in the subsequent year 2019 (9.87 ± 7.97, p < 0.01). *Anopheles fluviatilis* density was 0.17, 1.03 and 0.9 in 2017, 2018 and 2019, respectively.

The study found that 9 anopheline species were present in all categories (category A, category B, category C) areas (Table 2). The average per man hour anopheline density was slightly higher in category A (22.5). Similarly, the vector proportion was also found higher in villages of category A (63.4% *An. culicifacies* and 1.9% *An. fluviatilis)*, as compared to villages of category B (*An. culicifacies* 53.3 and *An. fluviatilis* 0.9%) and category C (*An. culicifacies* 49.7% and *An. fluviatilis* 4.1%). Statistically, the proportion of *An. culicifacies* was found significantly higher in category A (63.4%) when compared to category B (53.3%, p < 0.001) and category C (49.7%, p < 0.001). However, the difference between the category B and C villages was not statistically significant.

Overall, the proportion *An. culicifacies* and *An. fluviatilis* varied significantly from category A to category B and C (chi sq for linear trend = 30.74; p < 0.0001, Table 3) with significant variation in vector density among three category villages (F_2,153_ = 5.24; p < 0.01). The month-wise vector density was almost equal in all three areas throughout the year, except in January 2019 and July 2019 (Table 3).

**Fig. 2:**
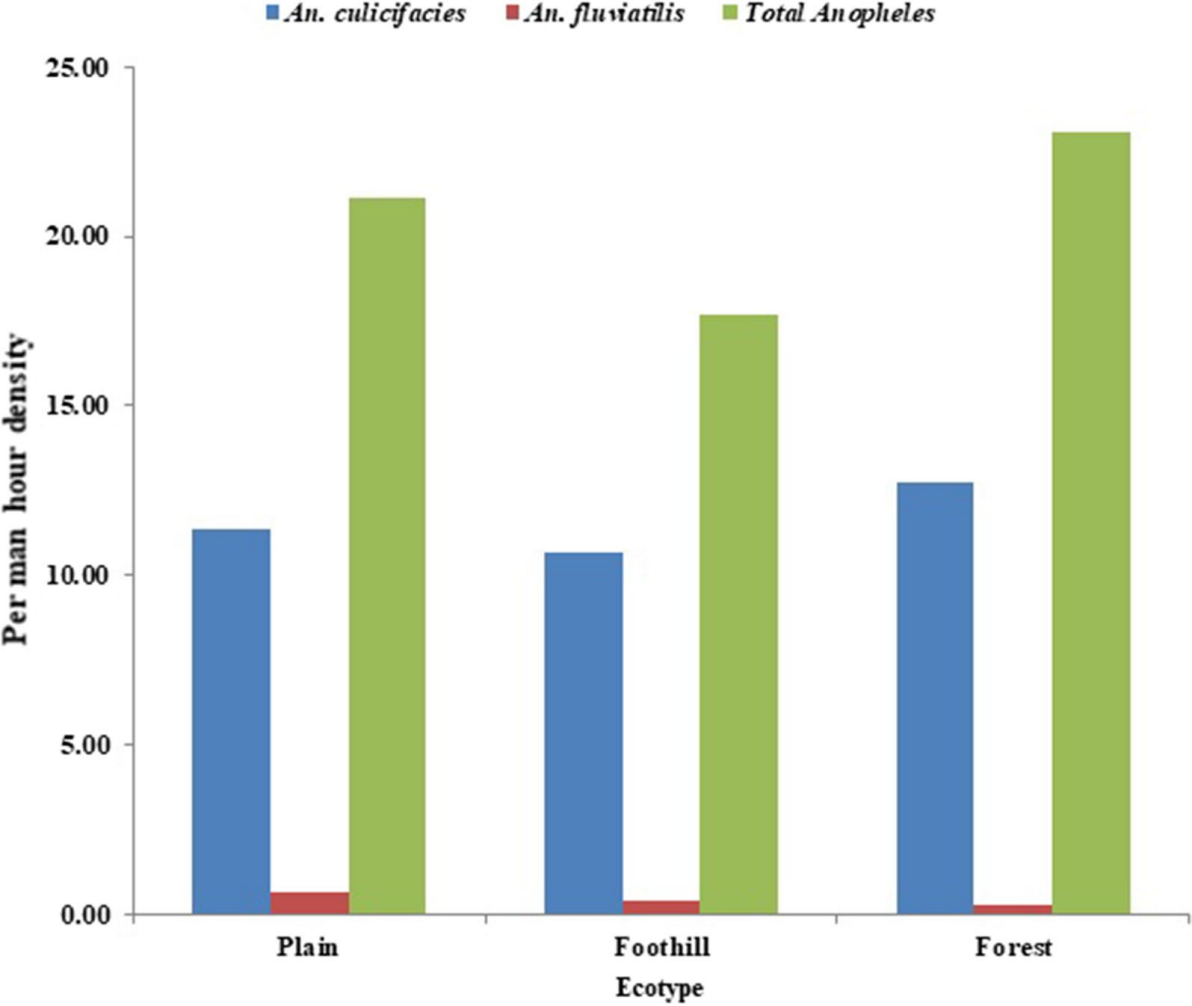
Ecotype wise indoor resting density of *An.culicifacies*, *An.fluviatilis* and anophelines

**Fig. 3:**
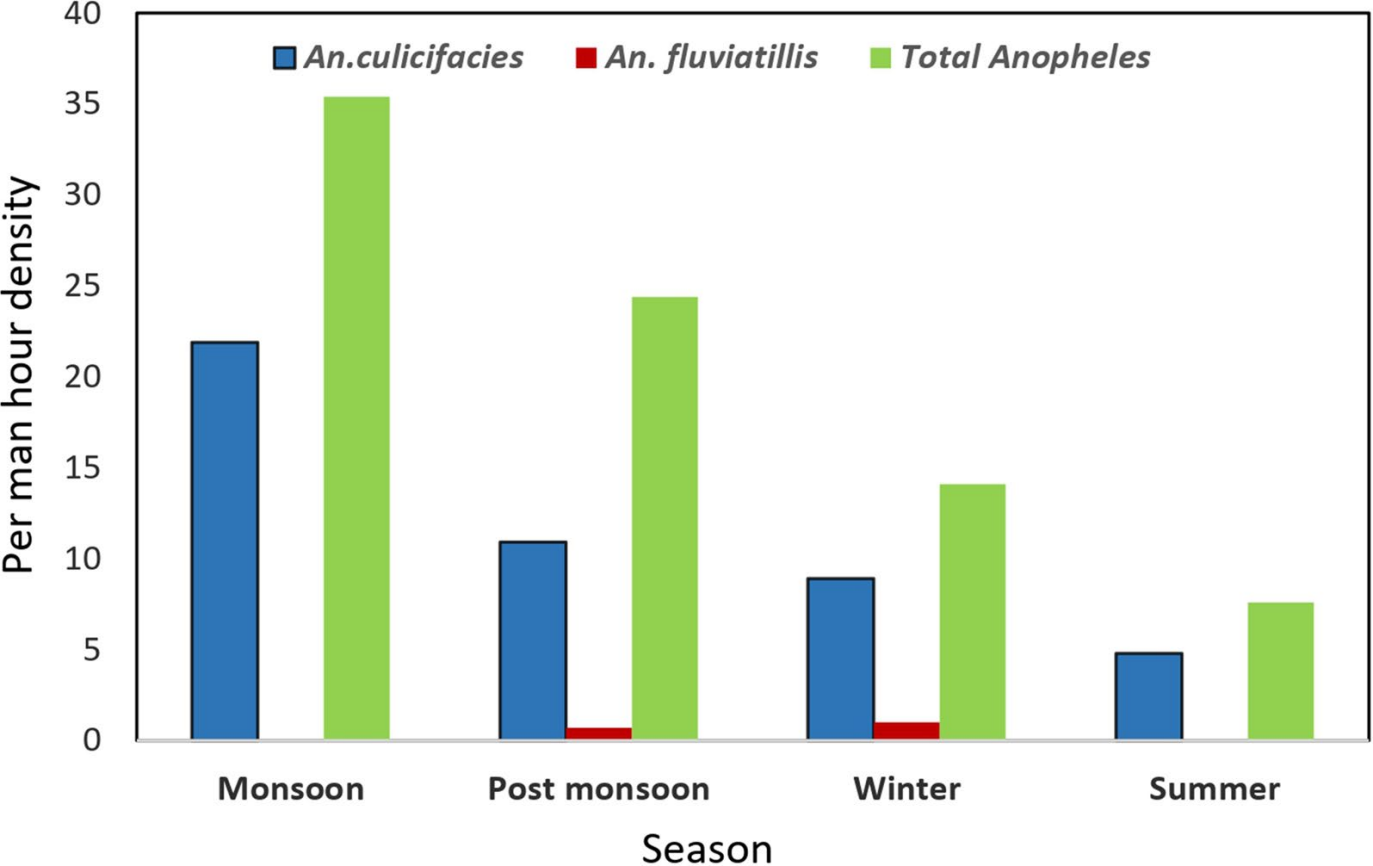
Season wise indoor resting density of *An.culicifacies*, *An.fluviatilis* and anophelines

The ecotype analysis revealed that the per man-hour density of anophelines and *An. culicifacies* was found almost equal in villages of plains (21.1 and 11.3) and forest (23.1 and 12.74). However, it was slightly lower in foothill villages (17.7 and 10.6) (Fig. 2). The highest per man hour density of anophelines was found in the monsoon season (35.4) followed by post monsoon (24.4), winter (14.1) and summer (7.6) (Fig. 3). The similar trend was found in *An. culicifacies* (21.9 in monsoon, 10.9 in post monsoon, 8.9 in winter and 4.8 in summer). However, *An. fluviatilis* slightly was higher in winter (1.0) as compared to post monsoon (0.7). *Anopheles fluviatilis* was not found in monsoon and summer season.

For mosquitoes caught resting, it was also observed that anopheline vector density was higher in cattle shed (20.7) as compared to the human dwelling (9.9) (Table 4). Most of the *An. culicifacies* (77.5%) and *An. fluviatilis* (73.2%) were caught from outside of the houses i.e., in cattle sheds. This trend was almost similar in all category of villages.

**Table 4:** Category wise mosquito density in Human dwelling and Cattle sheds in the Mandla district, Madhya Pradesh

Category of villages	Species	Human dwelling			Cattle sheds			Total in District	
		Nos	%	MHD	Nos	%	MHD	Nos	MHD
Category A (< 1 API)	*An. culicifacies*	156	21.00	6.00	587	79.00	22.58	743	14.29
	*An. fluviatilis*	4	18.18	0.15	18	81.82	0.69	22	0.42
	*Total Anopheles*	293	25.02	11.27	878	74.98	33.77	1171	22.52
Category B (1–5 API)	*An. culicifacies*	117	19.83	4.50	473	80.17	18.19	590	11.35
	*An. fluviatilis*	0	0.00	0.00	10	100.00	0.38	10	0.19
	*Total Anopheles*	249	22.51	9.58	857	77.49	32.96	1106	21.27
Category C (> 5 API)	*An. culicifacies*	133	28.12	5.12	340	71.88	13.08	473	9.10
	*An. fluviatilis*	15	38.46	0.58	24	61.54	0.92	39	0.75
	*Total Anopheles*	229	24.08	8.81	722	75.92	27.77	951	18.29
Total	*An. culicifacies*	406	22.48	5.21	1400	77.52	17.95	1806	11.58
	*An. fluviatilis*	19	26.76	0.24	52	73.24	0.67	71	0.46
	Total *Anopheles*	771	23.88	9.88	2457	76.12	31.50	3228	20.69

*Nos* Number, *MHD-*Per Man Hour Density, *API* Annual Parasite Incidence

### Insecticide resistance status of *Anopheles culicifacies*

The susceptibility tests were carried out in the month of October 2017 and October 2019. *Anopheles culicifacies* specimens were found resistant to DDT and malathion with mortality 24.8 to 28.0% and 50.5 to 84.0%, respectively. Possible resistance to alphacypermethrin was observed in October 2017 with 95% mortality. In the same year, susceptible to deltamethrin with 98.3% mortality was observed. However, in 2019, *An. culicifacies* specimens were found to be resistant to alphacypermethrin (82.5% mortality) with possible resistance to deltamethrin with 95.6% mortality (Table 5).

**Table 5:** Insecticide resistance status of *An. culicifacies* in the year 2017 and 2019 in Mandla district, Madhya Pradesh

Month	Insecticide	Replicates	Mosquito tested	Nos knocked down 1 h	Dead 24 h	% Mortality 24 h	Susceptibility status
October-17	DDT 4	5	75	16	21	28.0	R
	Malathion5%	5	75	58	63	84.0	R
	Alphacypermethrin 0.05%	8	120	92	114	95.0	PR
	Deltamethrin 0.05%	8	120	101	118	98.3	S
October-19	DDT 4	7	105	7	26	24.8	R
	Malathion5%	7	105	38	53	50.5	R
	Alphacypermethrin 0.05%	9	135	93	111	82.2	R
	Deltamethrin 0.05%	9	135	110	129	95.6	PR

*R* Resistance,* PR* Possible Resistance,* S* Susceptible,* DDT* Dichloro-Diphenyl-Trichloroethane

### Cone bioassays

**Fig. 4:**
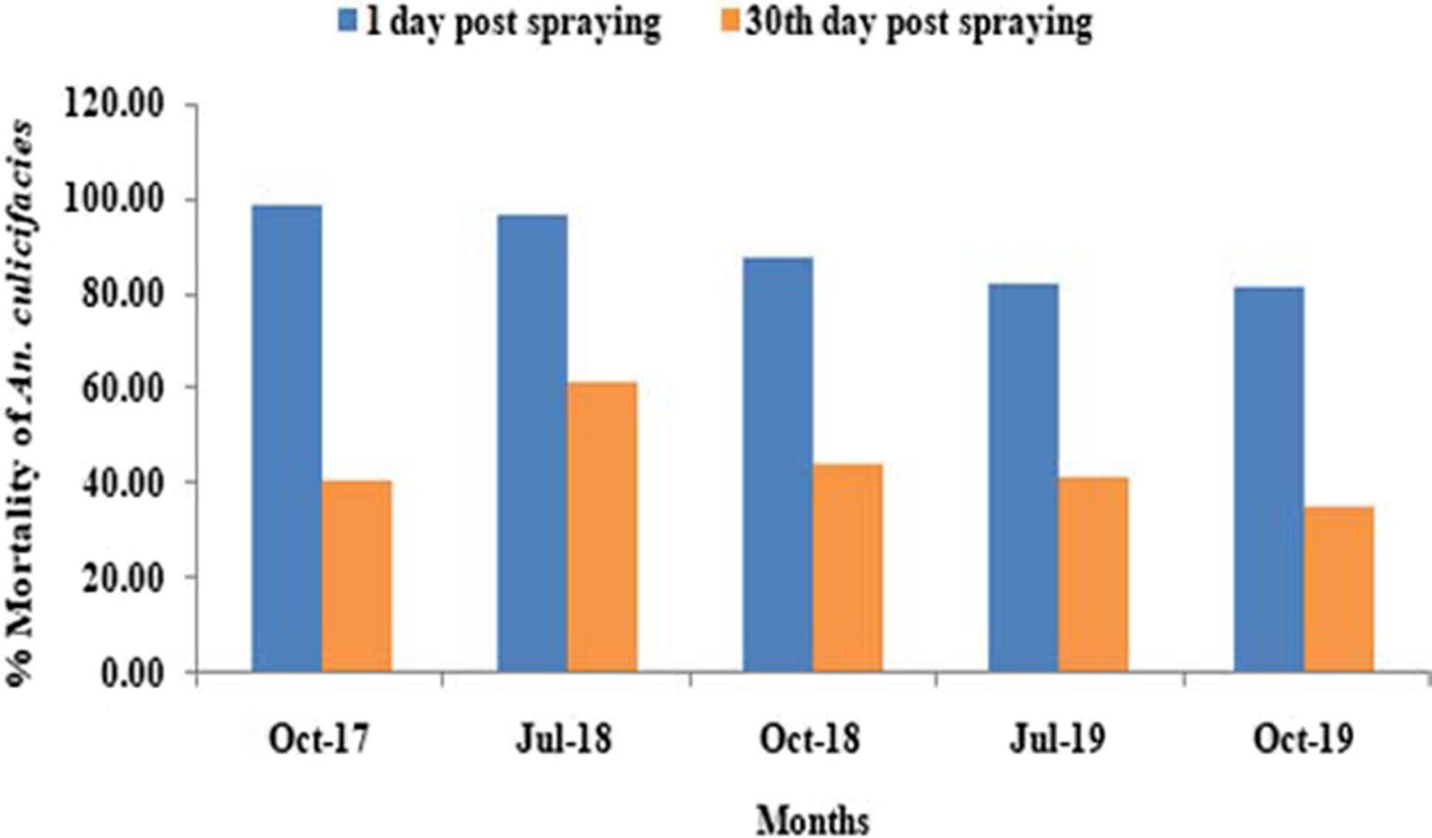
Cone bioassay showing the mortality percentage of *An.culicifacies*, on the day 1 and day 30 after the IRS in Mandla

The bioassays carried out on one-day post spraying revealed 98.7%, 96.7%, 87.3%, 82.1% and 81.2% average corrected % mortality of *An. culicifacies* in the month of October 2017, July and October 2018 and in July and October 2019, respectively (Fig. 4). The mortality data on day 30 after spraying was 40.6%, 61.7%, 44.4%, 41.4% and 35.0% for the same months/years.

**Fig. 5:**
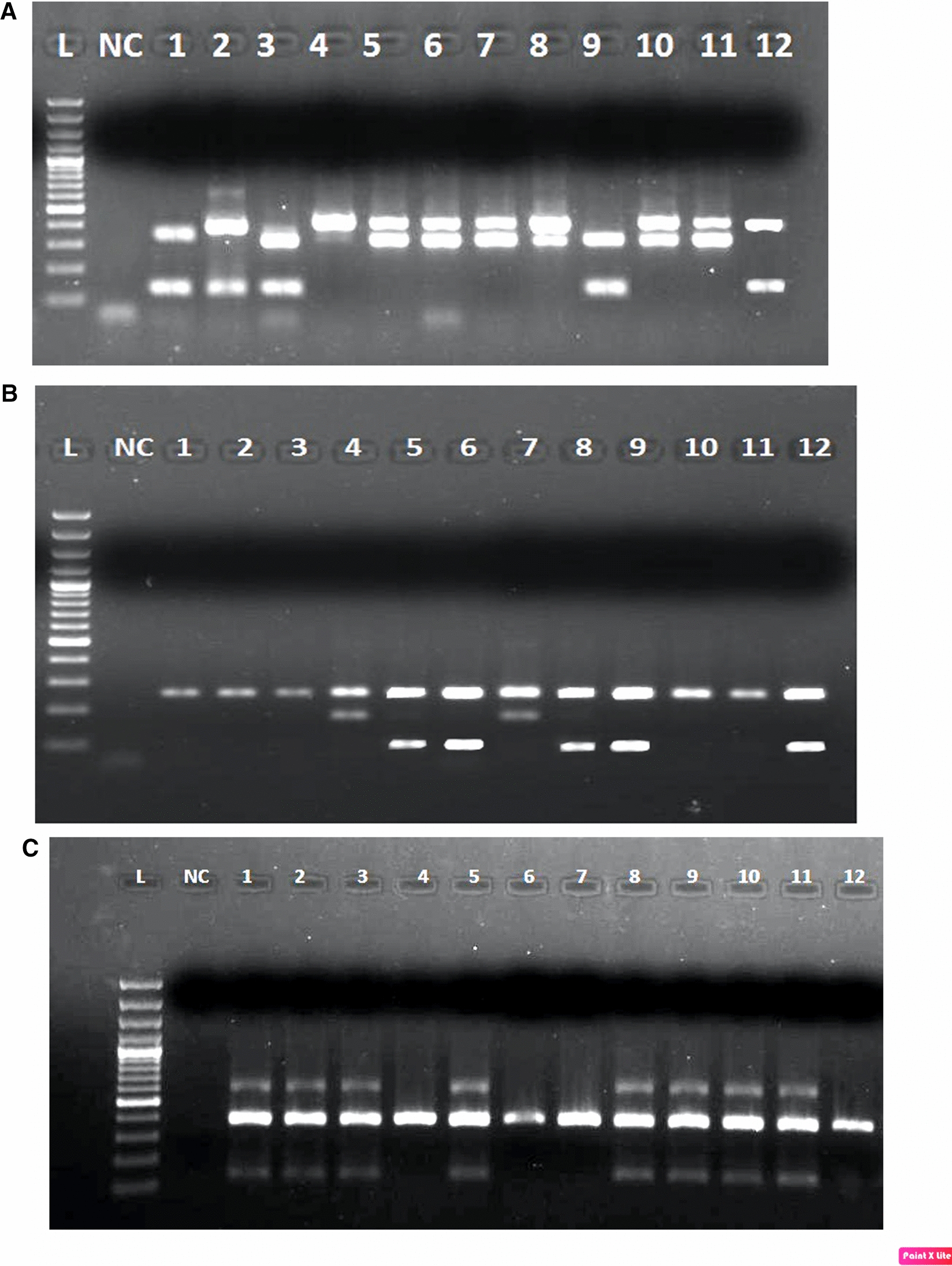
**a**: Gel image showing differentiation among *Anopheles culicifacies* in two groups; AD and BCE. Where L: 100 bp marker, NC: negative control, 1 to 12 samples positive for *Anopheles culicifacies* group. PCR product having double band from 1–3, 9 and 12 lane belongs to group BCE and 4 -8,10 and 11 lane belongs to AD group. **b**: Gel image showing differentiation among *Anopheles culicifacies* in B, C and E groups resp. Where L: 100 bp marker, NC: negative control, 1 to 12 samples positive for *Anopheles culicifacies* BCE groups. PCR products having single band from 1–3, 10 and 11 belong to B group, double band at 4 and 7 belong to E group and double band at lane 5, 6, 8, 9 and 12 lane belongs to C group. **c**: Gel image showing differentiation among *Anopheles fluviatilis* in S, T and U groups. Where L: 100 bp marker, NC: negative control, 1 to 15 samples positive for *Anopheles fluviatilis* group. PCR product having double band from 1–3, 5, 8–11 lane belongs to T group and 4, 6–7and 12 lane belongs to U group.

Vector incrimination by PCR*: *A total of 1806 *An. culicifacies* (743 from category A, 590 from villages of category B, and 473 from category C) and 71 *An. fluviatilis* (22, 10, and 39 from villages of category A, B and C), collected from both the human dwellings and cattle shed were processed for molecular detection of malaria parasites. One *An.culicifacies* specimen collected in the month of October 2019 from category A village was found positive for *Plasmodium falciparum* malaria parasites by ribosomal DNA PCR tests.

Sibling species identification: A total of 1,806 *An. culicifacies* were collected during the study period from different category villages. They were tested for sibling species determination. The PCR revealed the AD vs BCE group (Fig. 5a). The proportion of *An. culicifacies* C was the highest (38.5%) followed by A/D and E, and lowest was subspecies B (11.9%) (Fig. 5b, Table 6). The sibling species distribution was found almost similar in each area. However, *An. culicifacies* B was slightly higher (13.3%) in villages of category A than other two areas, whereas, *An. culicifacies* C was slightly higher (42.1%) in villages of category C than other two areas. *Anopheles culicifacies* A/D and E were found equally distributed in all the three areas. The *P. falciparum* positive, one *An. culicifacies* specimen was identified as sibling species C.

**Table 6:** Sibling species composition of *An.culicifacies* and *An.fluviatilis* in Mandla district, Madhya Pradesh

Village category	Season	*An. culicifacies* (n = 1806)					*An. fluviatilis* (n = 71)		
		B	C	E	A/D	Total identified	T	U	Total identified
Category A (< 1 API)	Rainy	70 (15.9)	144 (32.70	115 (26.1)	111 (25.2)	440	5 (41.7)	7 (58.3)	12
	Summer	15 (6.5)	96 (41.4)	61 (26.3)	60 (25.9)	232	4 (57.1)	3 (42.9)	7
	Winter	14 (19.7)	25 (35.2)	14 (19.7)	18 (25.4)	71	3 (100.0)	0 (0.0)	3
	Total	99 (13.3)	265 (35.7)	190 (25.6)	189 (25.4)	743	12 (54.5)	10 (45.5)	22
Category B (1–5 API)	Rainy	52 (12.6)	145 (35.1)	112 (27.1)	104 (25.2)	413	3 (60.0)	2 (40.0)	5
	Summer	8 (9.4)	40 (47.1)	16 (18.8)	21 (24.7)	85	0 (0.0)	0 (0.0)	0
	Winter	5 (5.4)	46 (50.0)	16 (17.4)	25 (27.2)	92	4 (80.0)	1 (20.0)	5
	Total	65 (11.0)	231 (39.2)	144 (24.4)	150 (25.4)	590	7 (71.0)	3 (30.0)	10
Category C (> 5 API)	Rainy	38 (12.8)	118 (39.6)	70 (23.5)	72 (24.2)	298	10 (71.4)	4 (28.6)	14
	Summer	7 (8.0)	45 (51.7)	14 (16.1)	21 (24.1)	87	0 (0.0)	0 (0.0)	0
	Winter	6 (6.8)	36 (40.9)	24 (27.3)	22 (25.0)	88	24 (96.0)	1 (4.0)	25
	Total	51 (10.8)	199 (42.1)	108 (22.8)	115 (24.3)	473	34 (87.2)	5 (12.8)	39
Total	Rainy	160 (13.90	407 (35.4)	297 (25.8)	287 (24.9)	1151	18 (58.1)	13 (41.9)	31
	Summer	30 (7.4)	181 (44.8)	91 (22.5)	102 (25.2)	404	4 (57.1)	3 (42.9)	7
	Winter	25 (10.0)	107 (42.6)	54 (21.5)	65 (25.9)	251	31 (93.9)	2 (6.1)	33
Grand total		215 (11.9)	695 (38.5)	442 (24.5)	454 (25.1)	1806	53 (74.6)	18 (25.4)	71

The terrain wise data revealed that all sibling species were present in all three terrains viz. plain, forest and foothill. Season-wise observations revealed that in each category of villages, all sibling species of *An. culicifacies* were present in all seasons. More number of B (13.9%), C (35.4%), E (25.8%) and A/D (24.9%) were found during monsoon/rainy season than the summers and winters. Out of 71 specimens of *An. fluviatilis* 53 (74.6%) were of species T (Fig. 5c, Table 6). The highest number (34) of T subspecies were detected from villages of category C. Season wise data revealed that in winters, 31 (93.9%) were of T, whereas in summers and rains, the proportions of T and U were almost the same (Table 6).

## Discussion

Malaria Elimination Demonstration Project (MEDP) is being undertaken in the Mandla district to demonstrate that malaria elimination is feasible using the existing case management and vector control tools and strategies. In the MEDP project, vector control is accomplished through the use of indoor residual spray and long-lasting insecticide treated nets and case management is accomplished by active surveillance combined with rapid diagnosis and prompt treatment [39].

This study has revealed that *An. culicifacies* and *An. fluviatilis* are present in the study area, which is in agreement with prior studies [9–12] in this region. The indoor resting densities of *An. culicifacies* was found throughout the year [9], possibly due to housing structures of tribal settlements. These structural characteristics of the houses, including cattle shed allow for easy mosquito ingress and egress and accordingly maintaining high densities.

However, in this study, less number of *An. culicifacies* were found inside the houses (human dwellings) as compared to the cattle sheds. This might be due to use of LLIN and application of IRS inside the houses. Prasad et al*.* [40] also reported a preference of mosquitoes to rest mainly in cattle sheds. With the increased distribution and enhanced use of LLIN in many areas, changes in vector behaviour from indoor resting to outdoor resting and from human dwelling to cattle sheds has also been observed in Odisha [41, 42]. The behaviour change was observed from human dwellings to cattle sheds in the study area. The *An. culicifacies* species transmit the disease mainly in rainy season i.e. July-October [43], which requires two rounds of IRS to be done to break malaria transmission cycle during the same period.

A prior study in the Balaghat district of Madhya Pradesh, which is adjacent to the Mandla district, has revealed that the insecticidal effect declined in one month after spraying of alphacypermethrin, which could be attributed to many factors such as improper spray, type of spray pumps, quality of insecticide, untrained man power and supervision [10]. In the present study, the results of the cone bioassay test also showed lower mortality of *An. culicifacies* one-month post spray during October 2017. Using the lesson learned from the Balaghat study [10] and present study observations in 2017, project implemented strict supervision program of the IRS campaigns in July 2018 using lessons learned from previous studies. Through the use of corrective protocols in IRS, study revealed a significant increase in mortality of *An. culicifacies,* which was remarkable, despite the observation of possible resistance against the used insecticides.

Subsequently in 2019, mortality of *An. culicifacies* declined in the study, which may be due to emergence of resistance against the alphacypermethrin insecticide. There is a need for a longitudinal entomologic study to get a full picture of the resistance pattern in the study area. The finding from this study that effective supervision of quality spray improves outcomes of spray further informs us that proper and well-supervised spray programmes would extend the life of insecticides and delay the emergence of resistance. Insecticide susceptibility status against the *An. fluviatilis* was not determined due to very low density in the study area for inadequate number of specimens. The lower vector density observed in the category C villages may be due to the impact of LLIN, because LLIN were introduced in these villages during the same time period.

*Anopheles culicifacies* have species complex of five sub-species with specific role in malaria transmission. In the present study, four species complex members of *An. culicifacies* species (B, C and E and A/D group) were found in the study area, of which C constituted about 38.5% in different collections followed by A/D, E and B. This observation is similar to the results of previous studies as the sub-species C considered efficient vector followed by A/D while sub-species B have least role in malaria transmission [6, 11]. The present study also found one specimen of *An*. *culicifacies* (sub-species C) infected with *P. falciparum* malaria parasites. The low number of sporozoite positivity is probably because of significant reduction in malaria cases in the study area, which would have significantly reduced the number of gametocytaemia positive cases thus not enabling infection of mosquitoes.

The sibling species T of *An. fluviatilis* was prevalent (74.6%) in the study area which is almost similar to the earlier studies carried out in Madhya Pradesh [11], where 99% *An. fluviatilis* were identified as T. In a study published in 2015, *An. culicifacies* C was found sporozoite positive along with *An. fluviatilis* T, which was previously known as non-malaria vector [11]. In Mandla, *An. culicifacies* E and *An. fluviatilis* T were not found in the earlier studies [6], while *An. culicifacies* E is very efficient malaria vector in India and also globally [16, 44, 45].

## Conclusion

Malaria vector control and elimination requires detailed knowledge of local vector species and their susceptibility to insecticides, as well as information on vector and human behaviours that may allow mosquitoes to avoid contact. Periodic collection of such data during elimination programmes is essential to inform vector control strategies and assess impact on malaria transmission. The current study provides information on entomological data collected during nine investigations which may be helpful in national malaria control and elimination programme.

## Data Availability

We have reported all the findings in this manuscript. The hardcopy data is stored at MEDP Office in Mandla, Madhya Pradesh and Indian Council of Medical Research-National Institute of Research in Tribal Health (ICMR-NIRTH), Jabalpur, Madhya Pradesh. Softcopy data is available on the project server of MEDP hosted by Microsoft Azure. If anyone wants to review or use the data, they should contact: Dr. Altaf A. Lal. Project Director–Malaria Elimination Demonstration Project, Mandla. Foundation for Disease Elimination and Control of India, Mumbai, India 482,003. E mail: altaf.lal@sunpharma.com.

## Abbreviations

API: Annual parasite incidence
DDT: Dichloro Diphenyl Trichloroethane
FDEC: Foundation for disease elimination and control
GMAP: Global malaria action plan
GTS: Global technical strategy
ICMR: Indian council of medical research
IEC: Institutional ethics committee
IRS: Indoor residual spray
LLINs: Long-lasting insecticide treated nets
MEDP: Malaria elimination demonstration project
NIRTH: National Institute of Research in Tribal Health
NVBDCP: National vector borne disease control programme
PCR: Polymerase chain reaction
PPP: Public–private-partnership
RBM: Roll back malaria partnership to end malaria
SEAR: South East Asian Region
SPSS: Statistical package for social sciences
